# Post-tracheostomy Bleeding: A Case Report on a Rare Variation of the Inferior Thyroid Artery

**DOI:** 10.7759/cureus.69242

**Published:** 2024-09-12

**Authors:** Hardip Gendeh, Nur Farahin Rosdi, Mohd Razif Mohamad Yunus

**Affiliations:** 1 Department of Otorhinolaryngology-Head and Neck Surgery, Faculty of Medicine, Universiti Kebangsaan Malaysia, Kuala Lumpur, MYS; 2 Department of Otorhinolaryngology-Head and Neck Surgery, Faculty of Medicine, Universiti Kebangsaan Malaysia, Universiti Kebangsaan Malaysia Medical Center, Kuala Lumpur, MYS; 3 Department of Otorhinolaryngology-Head and Neck Surgery, Universiti Kebangsaan Malaysia Medical Center, Kuala Lumpur, MYS

**Keywords:** common carotid artery, inferior thyroid artery, post-tracheostomy bleeding, thyroid artery, tracheostomy

## Abstract

This case report describes post-tracheostomy bleeding resulting from a rare occurrence of an aberrant course of the inferior thyroid artery (ITA), which arose directly from the common carotid artery (CCA). The patient, a 68-year-old male with severe COVID-19 complications, had undergone a tracheostomy due to prolonged intubation. Postoperatively, significant bleeding occurred from the tracheostomy site. Surgical exploration revealed the aberrant origin of the ITA. This case highlights the potential risks associated with aberrant vascular anatomy in the neck region.

## Introduction

This case report discusses a post-tracheostomy hemorrhage scenario, specifically delineating a rare anatomical variant of the inferior thyroid artery (ITA) that originates directly from the common carotid artery (CCA) instead of the thyrocervical trunk (TCT). The reported occurrence rate of the ITA originating from the CCA is approximately 1% or less [1,2]. The ITA normally originates from the TCT and loops behind the carotid artery, while the CCA does not usually have branches until it divides into the external and internal carotid arteries [1]. While the ITA generally follows a predictable course, undetected variations may present challenges such as unintentional injury and increased risk of complications, which include hemorrhage.

## Case presentation

A 68-year-old male presented with a severe bout of Category 5 COVID-19. His COVID-19 complications included right lung empyema with necrotizing pneumonia, bilateral pulmonary embolism, and acute kidney injury (AKI). The patient's critical condition required a multidisciplinary approach involving intensive care management and respiratory support. Due to high ventilation settings and the inability to be weaned off ventilatory support, he remained intubated for 15 days without a successful trial of extubation. The ventilator setting during the intensive care unit (ICU) stay was in pressure support intermittent mandatory ventilation (PSIMV) mode, with a positive end-expiratory pressure (PEEP) of 8 cm H₂O and the highest fraction of inspired oxygen (FiO₂) set at 60%. Therefore, a tracheostomy was indicated, and a timely referral was made to the otorhinolaryngology specialty.

Following consultation, the patient underwent the tracheostomy procedure successfully. The tracheostomy was performed under general anesthesia, with a horizontal incision made along the skin crease. The wound was explored layer by layer, and the strap muscles were retracted laterally. Intraoperatively, the trachea was centrally positioned, with a large thyroid gland present. The thyroid gland was released from its inferior attachment to the trachea and retracted superiorly. Once the trachea was identified, an incision was made, and a tracheostomy tube was inserted. The tube used was a cuffed, medical-grade polyvinyl chloride (PVC) tube, size 8.0 mm. Hemostasis was effectively secured using monopolar diathermy and applying absorbable hemostatic material, oxidized regenerated cellulose.

Twenty hours postoperatively, sudden bleeding was identified from the tracheostomy site, prompting immediate action from the anesthesia team. The anesthesia team applied compression with adrenaline-soaked gauze and noticed oozing from the left side of the tracheostoma. Ten minutes later, the patient went into cardiac arrest secondary to hypoxia and aspiration of blood clots. During attempts to bag the patient via the tracheostomy, challenges arose with the anesthesia team being unable to effectively bag via the tracheostoma, leading the anesthetist to opt for intubation. Cardiopulmonary resuscitation (CPR) commenced, and subsequently, return of spontaneous circulation (ROSC) was achieved. A size 8.0 mm endotracheal tube was successfully inserted with a single attempt aided by a video laryngoscope. Despite the discovery of a blood clot at the posterior commissure, the airway remained patent. To manage the ongoing bleeding, the tracheostomy tube was carefully removed, and continuous compression was applied.

**Figure 1:**
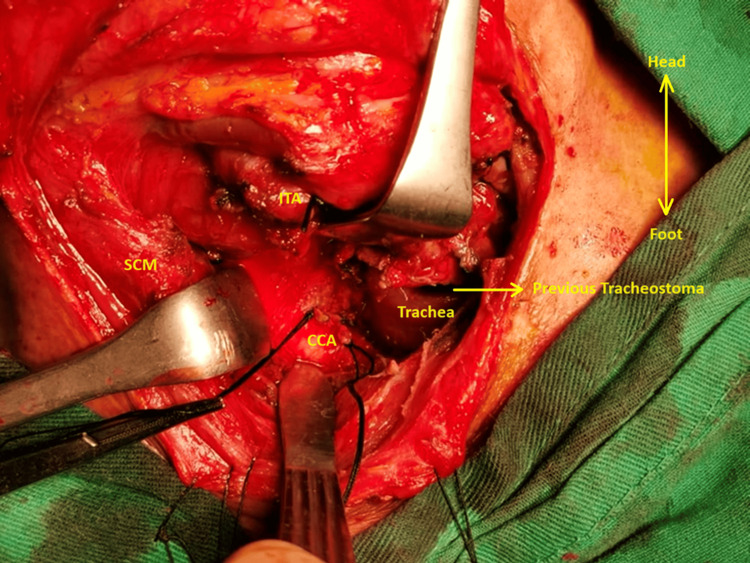
The inferior thyroid artery (ITA), ligated, with its branches originating from the common carotid artery (CCA) after retracting the sternocleidomastoid (SCM) muscle. Additionally, the ligated CCA branch is shown

The patient then underwent an examination under anesthesia and hemostasis for bleeding. Intraoperative findings revealed blood clots that bleed upon removal deep to the right sternocleidomastoid (SCM) muscle, where the source could not be visualized initially. Skin incision was extended to the right neck, anterior to the anterior border of the right SCM. The SCM muscle was then retracted and the omohyoid and infrahyoid muscles were released, following which the right internal jugular vein (IJV) and CCA were identified. This allowed the identification of the bleeding vessel, namely, the ITA, which was a direct branch of the CCA just inferior to the bifurcation. Anterior jugular veins (AJV) were thus meticulously tied bilaterally. Further examination of the tracheostoma revealed normal findings. As depicted in Figure 1, the anatomical structures and surgical interventions described in the case are shown.

The tracheostomy tube was reinserted, followed by the closing of the skin. Postoperatively, there were no further incidents of bleeding observed via the tracheostomy site and no complications such as hematoma. The patient passed away several weeks later due to COVID-19 complications. 

## Discussion

Tracheostomy is a surgical procedure used to establish direct airway access through the neck to the trachea, often employed in cases of prolonged mechanical ventilation and respiratory weaning. While beneficial, it carries risks such as bleeding, trauma to nearby structures including blood vessels and trachea, aspiration, and airway loss. Post-tracheostomy bleeding, an occurrence frequently caused by trauma or vascular injury, is a critical complication requiring immediate attention to secure the airway and prevent hypoxia and aspiration. Effective communication and prompt hemostasis are crucial in managing this condition effectively. Profuse bleeding can be caused by thyroid-innominate artery bleeding after one to two weeks.

Anatomically, post-tracheostomy bleeding is commonly associated with damage to the anterior jugular veins, innominate artery, or thyroid isthmus vessels, rendering it crucial to consider uncommon vascular anatomy as a potential cause [3]. In this case, an abnormal course of the ITA led to vulnerability during the tracheostomy procedure, resulting in bleeding.

**Figure 2:**
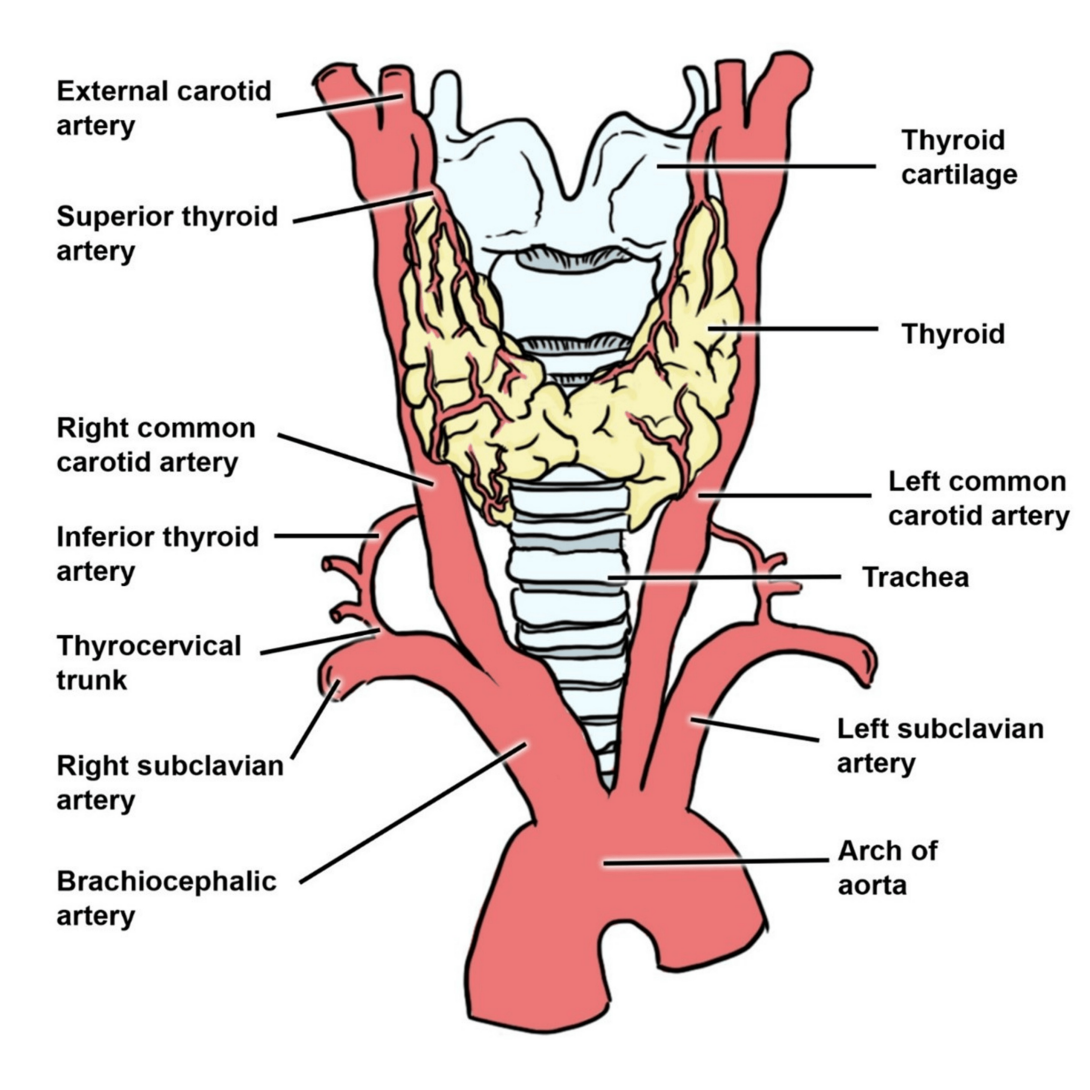
The anatomy of the inferior thyroid artery (ITA). It originates from the thyrocervical trunk (TCT), which, in turn, arises from the subclavian artery Image drawn by author

**Figure 3:**
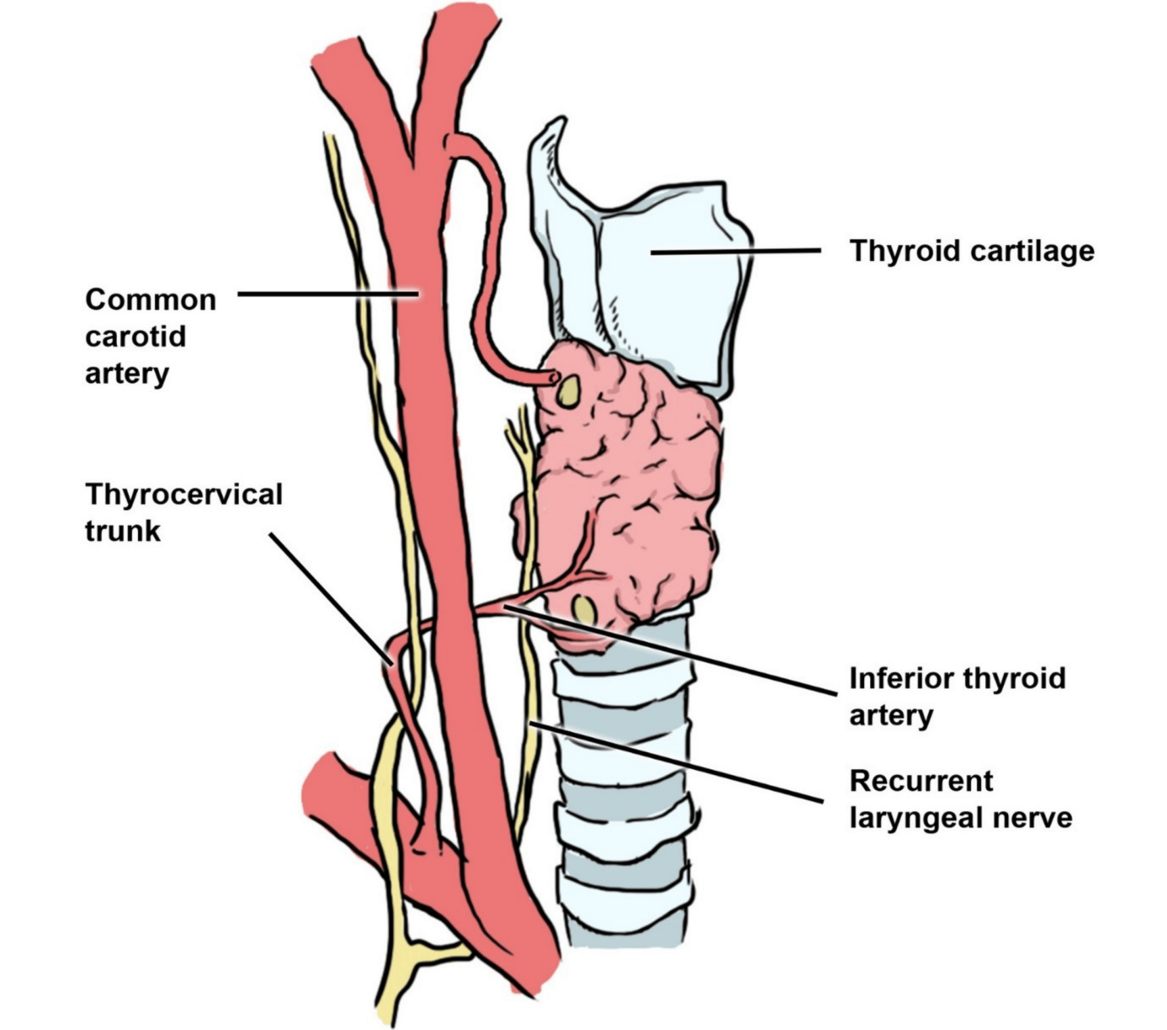
The anatomy of the inferior thyroid artery (ITA), right lateral view. It runs up superiorly and posteriorly to the carotid artery and divides into two or more branches when it reaches the middle of the thyroid gland. Contrarily, the common carotid artery (CCA) usually does not have any branches until it splits into the external and internal carotid arteries Image drawn by author

The ITA typically follows a predictable course as it supplies blood to the thyroid gland, normally originating from the TCT in 90.5% of cases [2]. The TCT, in which the ITA originates, arises from the subclavian artery. The ITA may also originate from the subclavian artery (7.5%), or in exceptionally rare cases, from the CCA (0.2%), aortic arch, brachiocephalic, internal thoracic, pericardiacophrenic, or vertebral arteries (0.6%) [2]. Figures 2-3 depict the ITA and its typical origin from the TCT accordingly.

In reference to the discussed case, the branching of the ITA from the CCA is uncommon. Although rare, such branching presents significant risks when carrying out surgical interventions in the area. Hemorrhage may potentially be induced and occur if the artery is inadvertently injured during an operation due to its unusual position and aberrant course [2]. The injury may be due to unintentional manipulation that caused the vessel to be lacerated or ruptured. Such risk is also commonly observed in other neck procedures such as thyroidectomy [2].

Another possible cause of hemorrhage is attributable to artery injury sustained following over-retraction and prolonged retraction of the thyroid gland superiorly, leading to stretching of the thyroid arteries, thus increasing the risk of vascular injury and subsequent bleeding. Anatomically, the thyroid isthmus overlies the first few tracheal rings, which often present as a barrier during tracheostomy [4]. Splitting of the isthmus is rarely necessary or warranted; even if it extends over the third ring, it can easily be dissected off the ring and retracted upward [5]. However, in this case, the thyroid gland was enormous, rendering its over-retraction possibly posing risks such as potential stretching of the thyroid arteries. Thus, in this case, consideration of thyroid split may be beneficial.

In a recent study, its findings imply that thyroid-split tracheostomy is comparably safe to the conventional thyroid-retraction tracheostomy [6]. Surgeons can choose between the two techniques based on their preference and the requirement for enhanced exposure and refined surgical manipulation of the thyroid gland. Therefore, identifying the thyroid gland with appropriate dissection away from the plane of surgery by either hooking it up or, in difficult cases, ligating and dividing the isthmus can help them reach the trachea. In cases involving a patient with a significantly enlarged thyroid, opting for thyroid-split tracheostomy may offer better outcomes.

## Conclusions

The ITA branching from the CCA is uncommon. Such an unusual position may lead to complications during surgery, potentially causing hemorrhage if the artery is injured due to its aberrant course. Consideration of alternative techniques, such as thyroid-split tracheostomy, and prevention of over-retraction may be beneficial in complex cases.
